# Chinese Few-Shot Named Entity Recognition and Knowledge Graph Construction in Managed Pressure Drilling Domain

**DOI:** 10.3390/e25071097

**Published:** 2023-07-22

**Authors:** Siqing Wei, Yanchun Liang, Xiaoran Li, Xiaohui Weng, Jiasheng Fu, Xiaosong Han

**Affiliations:** 1 Key Laboratory for Symbol Computation and Knowledge Engineering of National Education Ministry, College of Computer Science and Technology, Jilin University, Changchun 130012, China; weisq22@mails.jlu.edu.cn (S.W.); ycliang@jlu.edu.cn (Y.L.); xiaoran21@mails.jlu.edu.cn (X.L.); 2 Zhuhai Laboratory of Key Laboratory for Symbol Computation and Knowledge Engineering of Ministry of Education, Zhuhai College of Science and Technology, Zhuhai 519041, China; 3 School of Mechanical and Aerospace Engineering, Jilin University, Changchun 130012, China; wengxiaohui@jlu.edu.cn; 4 CNPC Engineering Technology R&D Company Limited, National Engineering Research Center of Oil & Gas Drilling and Completion Technology, Beijing 102206, China; fujsdr@cnpc.com.cn

**Keywords:** MPD, knowledge graphs, entity extraction, relation extraction, few shot

## Abstract

Managed pressure drilling (MPD) is the most effective means to ensure drilling safety, and MPD is able to avoid further deterioration of complex working conditions through precise control of the wellhead back pressure. The key to the success of MPD is the well control strategy, which currently relies heavily on manual experience, hindering the automation and intelligence process of well control. In response to this issue, an MPD knowledge graph is constructed in this paper that extracts knowledge from published papers and drilling reports to guide well control. In order to improve the performance of entity extraction in the knowledge graph, a few-shot Chinese entity recognition model CEntLM-KL is extended from the EntLM model, in which the KL entropy is built to improve the accuracy of entity recognition. Through experiments on benchmark datasets, it has been shown that the proposed model has a significant improvement compared to the state-of-the-art methods. On the few-shot drilling datasets, the F-1 score of entity recognition reaches 33%. Finally, the knowledge graph is stored in Neo4J and applied for knowledge inference.

## 1. Introduction

The demand for energy is increasing with global population growth and economic development. Oil and gas are major energy sources, crucial for national and regional economic development, energy security, technological innovation, employment opportunities, and enhancing a country’s status and influence. Therefore, oil and gas developments are of utmost importance. Drilling is a critical activity in oil and gas development, and managed pressure drilling (MPD) plays a key role in ensuring a safe and reliable drilling process while improving production efficiency. MPD is essential for safe work, resource protection, production efficiency, cost control, and environmental preservation. Effective MPD measures enable efficient, safe, and sustainable oil and gas development. In the process of using MPD for oil and gas development, a large amount of MPD field data have been generated, but these data are often disorganized and underutilized. Constructing a knowledge graph of the MPD domain can improve the current state of disorganized data and enhance their accessibility. Hence, this paper focuses on constructing a knowledge graph based on the aforementioned study. The construction of a knowledge graph involves fundamental steps such as knowledge extraction, knowledge fusion, and quality control. Entity extraction and relationship extraction represent typical knowledge extraction tasks.

Named entity recognition (NER), also known as entity extraction, is primarily focused on identifying the text range of entities and categorizing them into predefined categories. Four common methods are employed for NER.

**Rule-based methods**: These methods utilize manually written rules and patterns to identify named entities. While simple and easy to implement, they require extensive manual effort and are not suitable for complex entity recognition tasks.

**Dictionary-based methods**: This approach utilizes preconstructed dictionaries or knowledge bases to match entities in text. It offers the advantage of speed and high customization but fails to recognize entities not present in the lexicon.

**Machine learning-based methods**: Machine learning algorithms, such as conditional random fields (CRF), support vector machines, maximum entropy Markov models, and deep learning models, are employed to learn and predict named entities in text. These methods require well-labeled training data for effective model training and have the capability to automatically learn features and patterns for various entity recognition tasks.

**Deep learning-based methods**: Deep learning-based models, such as long short-term memory networks (LSTM), have achieved significant advancements in NER. These models capture contextual information and semantic features in text. With large-scale labeled training data, they automatically learn and extract features, leading to successful NER. In recent years, deep learning-based methods have become mainstream approaches, exhibiting significant performance improvements in NER tasks. For example, Dai et al. [1] employed a BERT-BiLSTM-CRF architecture to extract named entities from Chinese electronic medical records.

Few-shot NER addresses the challenge of data scarcity in NER tasks. Traditional approaches require a large number of labeled samples to train the model effectively. However, practical applications often involve domain-specific or type-specific named entities with limited sample data, posing a challenge for model training. Few-shot NER aims to address this challenge [2,3,4,5,6]. Common methods include transfer learning, meta-learning, active learning, data augmentation, and distant supervision. Prompt-based learning has recently achieved significant success in few-shot classification tasks. The success of prompt-based learning depends on two factors: (1) reusing masked language model targets to bridge the gap between pre-training and fine-tuning targets [7,8,9]; and (2) designing complex templates and labeled words to help the language model adapt to task-specific answer distributions, thereby improving performance with fewer samples. Manual selection [2,3], gradient-based discrete search [10], language model generation [11], and continuous optimization [12] are popular approaches used to induce appropriate answers in corresponding tasks.

However, template-based prompt tuning is primarily designed for sentence-level tasks and is challenging to adapt to token-level classification tasks like NER. The search for suitable templates becomes more difficult as the search space expands in NER, and relying on a small number of annotated samples can lead to overfitting. To address this issue, the Chinese EntLM with new loss (CEntLM-KL) model inspired by EntLM for Chinese few-shot entity extraction (CEntLM) is presented in this paper. The proposed model formulates the NER task as a language model (LM) problem without templates and is further applied to entity extraction, combined with the results of entity extraction based on Trie trees [13].

Entity relationship extraction (ERE) aims to extract semantic relationships between entities from text. It is a crucial task in natural language processing and information extraction, facilitating the understanding of connections between entities in a text and supporting various applications. ERE methods include rule-based extraction, supervised learning, distant supervised learning, semi-supervised learning, and transfer learning. In this paper, we adopt a rule-based method for relationship extraction in the construction of the knowledge graph, as it offers higher accuracy and simplicity. Rule formulation and relationship extraction are performed based on subject terms, entity location relationships, and keywords.

The main contributions of this paper are as follows.

Firstly, a novel model, CEntLM-KL, is proposed for Chinese few-shot NER. Given the presence of domain-specific or type-specific named entities in practical applications with limited sample data, a novel model, CEntLM-KL, is proposed for Chinese few-shot NER. Inspired by the EntLM [14] model, the CEntLM-KL model formulates the NER task as an LM problem without relying on templates. The model demonstrates good performance on both private and public datasets, including the People’s Daily-NER Dataset and the few-shot dataset.

Secondly, in this paper, we complete the structure of the MPD domain knowledge graph, defining 14 entity types. Based on an analysis of the specific requirements of the MPD domain, structure of the Knowledge Graph design is completed, defining 14 types of entities (e.g., well class, well type, drilling fluid type, complex working conditions, performance of complex working conditions, handling measures for complex working conditions, and possible causes of complex working conditions) and eleven types of relationships (e.g., well class−drilling fluid type).

Finally, a knowledge base is built for the MPD domain in this paper. A domain knowledge base is built by crawling the Internet and CNKI related literature as well as collating relevant owned MPD data, including a structured knowledge base, a manually constructed domain dictionary based on the acquired knowledge, and a literature base consisting of published papers and drilling reports. Entity extraction and relationship extraction are performed sequentially using knowledge extraction in the data layer construction. For entity extraction, the unstructured text is annotated with BIO by the constructed domain dictionary to generate a labeled entity dataset. The relationship extraction uses a rule-based approach to ensure high accuracy and simplicity. Rule formulation and relationship extraction are performed based on subject terms, entity location relationships and keywords.

## 2. Methods

**Figure 1:**
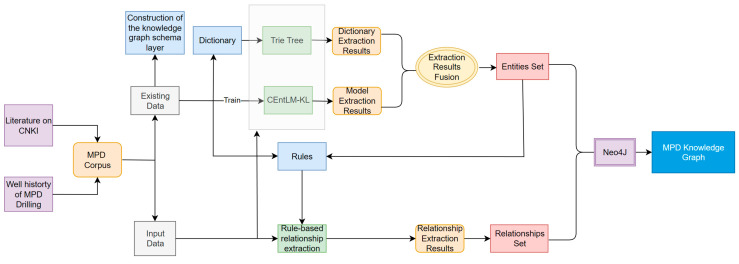
Pipeline of our work.

The main goal of this paper is to improve the template free NER model EntLM, and establish the MPD knowledge graph. In the process of constructing the knowledge graph, the first step is to use the existing data to construct the knowledge graph schema layer to obtain the entity types and relationship types required to construct the MPD knowledge graph. Then, the improved model CEntLM-KL is trained using the existing data, and the trained deep learning model is combined with the Trie tree method to extract entities from the input data. A rule-based method is used to extract relationships from the input data. Finally, Neo4J is used to store the obtained entities and relationships and construct a visual Knowledge graph. The overall framework of the paper is shown in Figure 1.

### 2.1. Template-Based Tuning

Template-based tuning is a technique used to optimize and fine-tune LMs by combining template design and fine-tuning approaches. In this technique, a template is designed with keywords and placeholders to guide the generation task. The LM is then fine tuned using input samples that contain these templates, allowing it to better understand and generate text that aligns with the template.

By employing template-based tuning, the LM can generate text that adheres to the template, resulting in improved quality and consistency of the generated output. This technique has potential applications in various natural language processing tasks, including text generation [15], dialogue systems [16], question-and-answer systems [17], recommendation system [18], and text clustering [19]. It can enhance the accuracy, coherence, and usefulness of the generated results, thereby improving the overall performance and robustness of the model.

However, when applied to NER, the template-based approach becomes more complex. In NER, the goal is to obtain a sequence of tokens corresponding to each character in the input. Therefore, an additional placeholder, denoted as [*S*], needs to be added to the constructed template to accommodate the characters or character sequences. For example, the template for an LM model could be “[*X*][*S*] is a [*Z*] entity,” and during training, the model predicts the labeled words at the [*Z*] position.

During the decoding process, to obtain the label sequence for a given input, all possible spans of the input sequence must be enumerated and considered.

(1) $$Y=\left\{\begin{array}{c} \mathrm{ar}gmax\;P\left(\left[Z\right]=M\left(Y\right)|T_{prompt}\left(X,s_{j}^{i}\right)\right),\\ s_{j}^{i}=Enumerate\left(\left\{x_{i},\ldots \ldots ,x_{j}\right\},i,j\in \left\{1,n\right\}\right) \end{array}\right\}$$

where *T_prompt_* is a function that converts input *X* into prompt input, and function *M* is a mapping function that connects the label word *Y* mapping to the label space.

**Figure 2:**
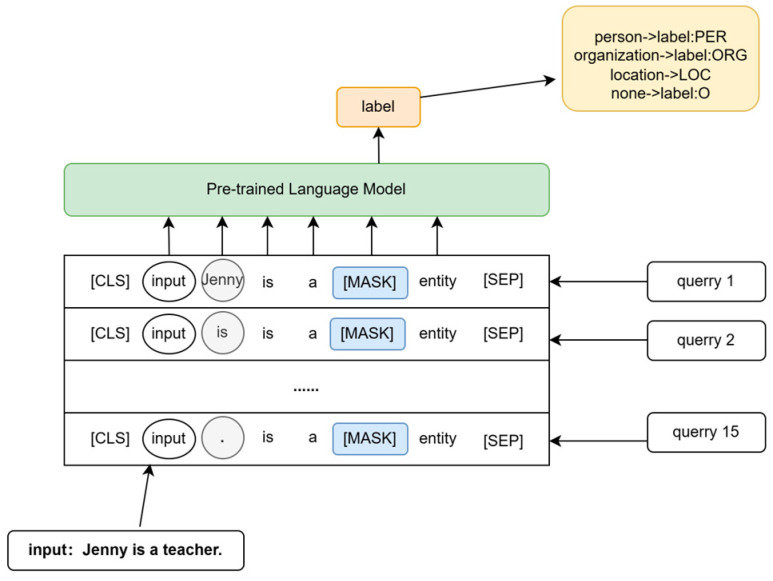
Example of NER’s template-based prompt method.

As shown in Figure 2, let us consider an example with the phrase “Jenny is a teacher.” In this case, the model would need to enumerate 15 times to obtain the desired label sequence. This decoding process is time consuming, and the decoding time varies depending on the length of the input sequence. Consequently, while the template-based cue fine-tuning method is highly effective for small samples, it is not suitable for the NER task due to its inefficiency.

### 2.2. Chinese Entity-Oriented LM Fine Tuning

**Figure 3:**
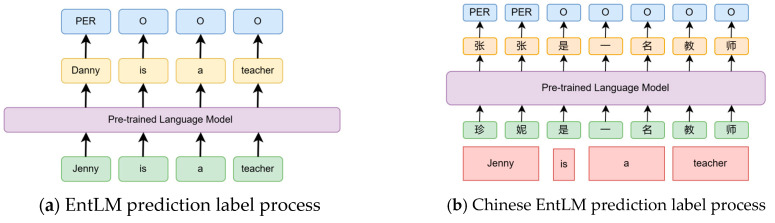
Comparison of the flow of Chinese and English EntLM prediction labels.

The EntLM method introduces an innovative approach to template-free NER while leveraging the benefits of prompt tuning. Instead of using templates to guide the LM during fine tuning, this work proposes a novel objective called the entity-oriented LM goal, the flow is depicted in Figure 3a. During the prediction of the label sequence “Jenny is a teacher.”, the LM is trained to predict “Jenny” as “Danny”. Here, “Danny” serves as an indicator for the “PER” label, and indicators for other labels are determined using the label selection algorithm described below. Non-entity words are predicted as the original words. In this paper, we focus on Chinese entity-oriented LM fine tuning, and the process is illustrated in Figure 3b. For an input sentence “珍妮是一名教师”, which means “Jenny is a teacher”, the target sentence is obtained by replacing the corresponding position of the entities in the input sentence with the label word, while the other positions remain unchanged. Here “张” represents the “PER” label. It is a very common surname in China. The corresponding position character remains unchanged to represent the “O” label.

### 2.3. Label Word Generation

For the labeled data, there are three methods of label word selection.

#### 2.3.1. Searching with Data Distribution

To identify the labeled words in a specific class, we employed a frequency-based approach on the corpus. Specifically, we calculated the occurrence frequency of each word ‘*w*’ within Class C individually using $\phi \left(x=w,y^{*}=C\right)$. Then, we ranked the words based on their frequencies and selected the most frequently occurring words as the labeled words for that particular class.

(2) $$M\left(C\right)=arg\underset{w}{max}\phi \left(x=w,y^{*}=C\right)\;$$

#### 2.3.2. Searching Using LM Output Distribution

In this method, a pre-trained LM is employed to search for label words. First, each sample $\left(X,Y^{*}\right)$ is input to LM to obtain the probability distribution $p\left({\hat{x}}_{i}=w|X\right)$ of each word *w* ∈ *V* (*V* is the vocabulary) at each position. Suppose $I_{top_{k}}\left({\hat{x}}_{i}=w|X,Y^{*}\right)\to \left\{0,1\right\}$ is used to determine whether w belongs to the top-k prediction of the sample. Finally, the labeled words of Class C can be obtained by Formula (3).

(3) $$M\left(C\right)=arg\underset{w}{max}\overset{\left|X\right|}{\underset{i}{\sum }}\phi_{top\_k}\left({\overset{\wedge }{x}}_{i}=w,y^{*}=c\right)\;\;\;$$

where $\phi_{top\_k}\left({\overset{\wedge }{x}}_{i}=w,y^{*}=C\right)=I_{top\_k}({\overset{\wedge }{x}}_{i}=w|X,Y^{*})\cdot I\left(y_{i}^{*}=C\right)$ denotes the frequency of *w* appearing in the top-k predictions of Class C.

#### 2.3.3. Search Using Both Data and LM Output Distributions

Considering both data and LM output to select label words, the label words of Class C are obtained by the following equation.

(4) $$M\left(C\right)=arg\underset{w}{max}\left\{\underset{\left(X,Y^{*}\right)\in D}{\sum }\overset{\left|X\right|}{\underset{i}{\sum }}\phi \left(\overset{\wedge }{x_{i}}=w,y_{i}^{*}=C\right)\cdot \underset{\left(X,Y^{*}\right)\in D}{\sum }\overset{\left|X\right|}{\underset{i}{\sum }}\phi \left(\overset{\wedge }{x_{i}}=w,y_{i}^{*}=C\right)\right\}$$

#### 2.3.4. Removing Conflicting Label Words

Because the high-frequency label words selected during the label word selection process may overlap across different categories, using them directly in the final training may lead to conflicts. To address this issue, a step is taken to remove conflicting label words for Class *C*. Th is the manually set threshold value. *w* is used as a label for category *C* only if the ratio of the frequency of the word *w* in category *C* to the frequency of the *w* in all categories is greater than *Th*. The specific procedure for removing conflicting label words is as follows:

(5) $$w=M\left(C\right),if\frac{\phi \left(x=w,y^{*}=C\right)}{\sum_{k}\phi \left(x=w,y^{*}=k\right)}>Th$$

In order to utilize more information about the label words, a virtual label word approach can be used, where the virtual label word for each class uses the average vector of high frequency words for each class as a prototype.

### 2.4. CEntLM-KL Model

**Figure 4:**
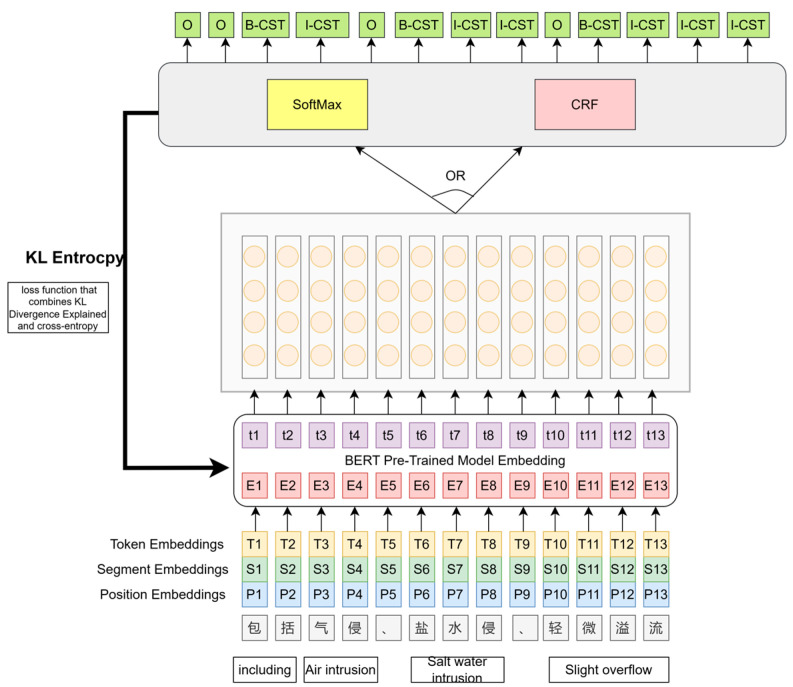
Overall architecture of CEntLM-KL.

For a given input sentence *X* = {$x_{1}$*,*$x_{2}$*,...,*$x_{n}$} and its corresponding label sequence *Y = {*$y_{1}$*,*$y_{2}$*,...,*$y_{n}$*}*, after the input sequence is processed by the fine-tuned BERT, the model utilizes two decoding methods: Softmax and CRF. The final target sentence is obtained by replacing the entity positions in the input sentence *X* with the label words, while keeping the tokens at the other positions unchanged, i.e., *X* = {$x_{1}$*,*$x_{2}$*,...,M*$\left(y_{i}\right)$*,...,*$x_{n}$}. The overall architecture of the model is depicted in Figure 4, which illustrates the different components and their connections within the model. The BERT is the pre-trained Chinese-BERT.

In general, the loss function for the task of NER is in the form of cross-entropy, as shown in Formula (7). In order to make the probability distribution obtained from the model prediction closer to the distribution of the real results, we choose to use a loss function that combines Kullback−Leibler Divergence Explained and cross-entropy and we named it the KL entropy. So, the loss function of the model is set as Formula (6).

(6) $$L_{CEntLM-KL}\;=e^{-L_{EntLM}\;}\left(L_{EntLM}\;+a*log(1-\left(e^{-L_{EntLM}\;}{)}^{\frac{1}{a}}\right)\right)$$

(7) $$L_{EntLM}=-\overset{n}{\underset{i=1}{\sum }}logP\left(x_{i}=x_{i}^{Ent}|X\right)$$

where *a* is a hyperparameter, which is set to 1944 in this model. $p\left(x_{i}=x_{i}^{Ent}|X\right)=Softmax\left(W_{lm}.h_{i}\right)$.$\;W_{lm}$ are also the parameters of the pre-trained LM head. No new parameters are introduced in the proposed approach and it avoids the need for complex template construction in the NER task. This allows us to maintain the capability of handling tasks with limited labeled samples, as observed in cue-based approaches. During testing, the test input *X* is fed directly into the model, and the probability of labeling characters with Class *y* ∈ *Y* is modeled as Formula (8).

(8) $$p\left(y_{i}=y|X\right)=p\left(x_{i}=M\left(y\right)|x\right)$$

Only one decoding process is needed to obtain all tokens for each sentence, which is more efficient than the template-based prompt task.

### 2.5. Construction of the Knowledge Graph Schema Layer

**Figure 5:**
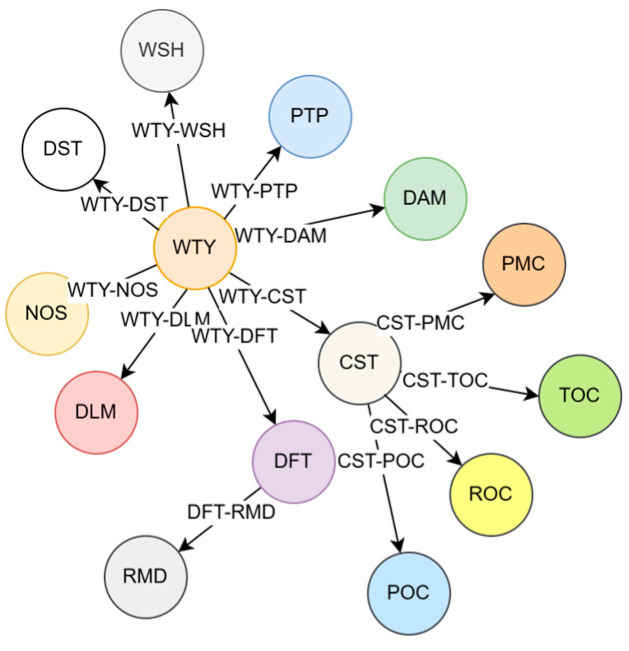
Various types of relationships between entities.

Before constructing the knowledge graph, it is crucial to understand the specific requirements of the drilling well control domain. Through careful analysis and investigation, a comprehensive list of 14 major categories of entities and 11 categories of relationships is identified. These entities and relationships are presented in Table 1 and Figure 5, respectively, providing a clear overview of the domain-specific entities and their interconnections. Based on these relationships, it can be seen that there are more relationships related to the types of wells (WTY) and complex working conditions in the drilling (CST), indicating that WTY and CST are two very important entity types in the process of MPD.

### 2.6. Entity Extraction Based on Fusion Model

**Figure 6:**
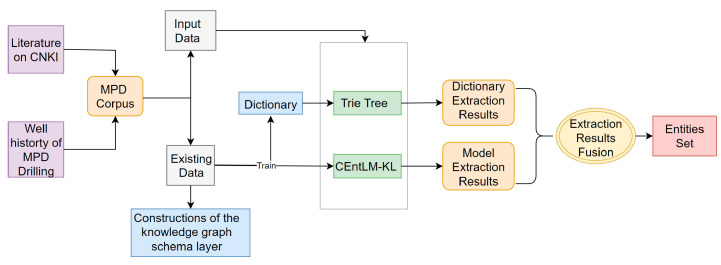
Fusion entity extraction process.

This paper proposes an entity extraction framework that combines the Trie tree method and the CEntLM-KL model. The framework, depicted in Figure 6, involves two rounds of entity extraction.

**Figure 7:**
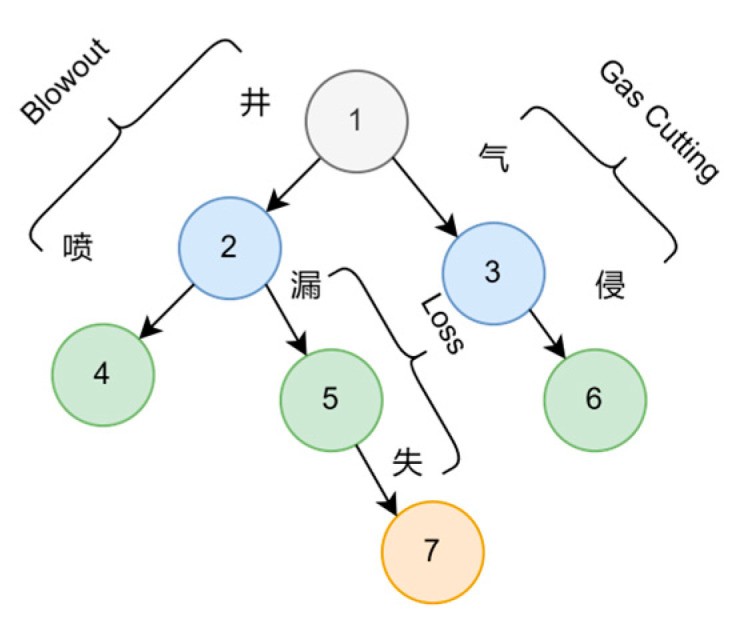
Trie tree example.

In the first round, Trie trees are constructed based on a manually constructed dictionary derived from existing data. Figure 7 provides an example of a Trie tree. This dictionary primarily captures the known entities in the domain. Once the Trie tree is obtained, the next step is to extract entities and annotate the data using the BIO scheme. For documents such as literature, the following steps are performed after sentence segmentation.

Candidate Word Generation: Algorithm 1 is executed to generate candidate words from the input text. This algorithm identifies potential entities by considering different combinations of consecutive words within each sentence.

Trie Tree Matching: The generated candidate words are then matched against the Trie tree. If a candidate word successfully matches an entity in the Trie tree, it is annotated with the corresponding BI (Beginning or Inside tag) to indicate the entity boundary. If a candidate word does not match any entity in the Trie tree, it is annotated with the O tag (Outside) to indicate that it is not part of any entity.

By annotating the data using the BIO scheme, each word in the input text is assigned a specific tag indicating its role in the entity extraction task. This annotation process helps in training and evaluating the performance of entity extraction models. The Trie tree method is then applied to extract entities from the input data and to generate a dataset for the subsequent deep learning model.

The second round of entity extraction utilizes a trained deep learning model. The model is trained on the dataset obtained from the first round and focuses on extracting potential entities that were not discovered by the Trie tree method. The extraction results from this round are subsequently merged with the first round’s results to obtain a comprehensive entity set.

**Algorithm 1:** **Find Entity****Input**: root: Node, sentence: String **Output**: entities: List
1:  curNode ← root; index ← 0; maxLength ← 10; entities ← [ ]
2: **while** index < len(sentence) **do**:
3:  j ← maxLength
4:  **while** j ! = 0 **then**
5:   word ← sentence [index: index + j]
6:   **if SeachWord**(root, word) = **True then**
7:        index ← index + j − 1; entities.add(word);
8:   **end if**
9:   j ← j − 1
10:   **end while**
11:   index ← index + 1
12: **end while**
13: **return** entities

### 2.7. Rule-Based Relationship Extraction

In this paper, a rule-based method is employed for relationship extraction. The method leverages the analysis of a significant amount of text data to identify patterns and expressions commonly used to indicate relationships. Based on this analysis, matching rules are formulated to extract relationships from the text. The main methods for rule formulation are as follows.

1. Entities Word-Based Extraction: This method focuses on identifying relationships based on the subject words involved. By examining the subject words and their corresponding contexts, relationships between entities can be inferred. Matching rules are defined to capture specific patterns and linguistic cues indicating relationships.
2. Keyword-Based Extraction: This method relies on the presence of certain keywords or key phrases that frequently co-occur with specific relationships. By identifying these keywords and using them as indicators, relationships can be extracted. Matching rules are designed to detect the occurrence of these keywords in the text and associate them with the appropriate relationships.

In this paper, two methods, namely Entities Word-Based Extraction and Keyword-Based Extraction, are selected for relationship extraction. Table 2 shows the types of relationships based on entity word extraction and the entity words required to extract the relationships. Table 3 shows the types of relationships based on keyword extraction and the keywords required for extracting the relationships.

## 3. Experiment and Results

### 3.1. Datasets

**Figure 8:**
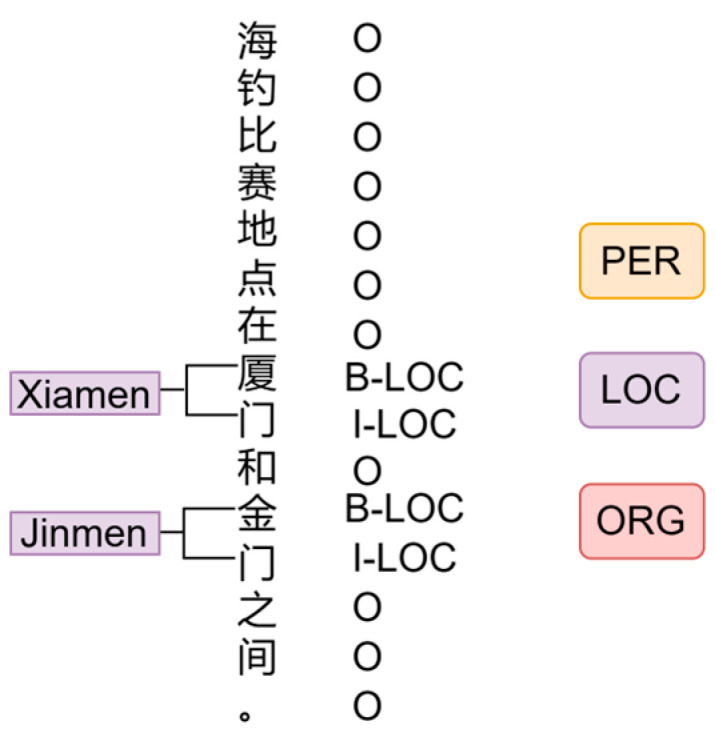
Example of BIO labeling.

There are three labeled datasets for NER, all using BIO for labeling. Figure 8 shows an example of BIO annotation for ‘海钓比赛地点在厦门和金门之间’ which means ‘The sea fishing competition is between Xiamen and Jinmen.’ The first one is the People’s Daily-NER Dataset. This NER dataset contains 3 common entity types of Person Name (PER), Place Name (LOC), and Organization Name (ORG). The second one is the Few-shot-NER Dataset. A 10-shot dataset extracted manually from the above People’s Daily-NER dataset. The numbers of entities in this dataset labeled as PER, LOC, and ORG types are all 10. The final dataset is the MPD Dataset which is a domain knowledge dataset that was established by collecting 150+ documents obtained by crawling the Internet and CNKI related materials, as well as collating our own, relevant, 20+ MPD well history materials. The dataset contains 14 types of entities as shown in Table 1 above. The composition of the datasets is shown in Table 4.

### 3.2. Comparison Experiment

Experiments were conducted using the three datasets described above.

To establish a baseline for comparison, four baseline models were selected. These baseline models represent existing approaches or techniques in the field of entity extraction and relationship extraction. The results obtained by these baseline models are presented in Table 5.

Table 5 showcases the comparative performance of the proposed model against the baseline models.

The results presented in Table 5 serve to highlight the strengths and improvements of the proposed model in comparison to existing approaches. They provide quantitative evidence of the model’s effectiveness and its potential contributions in the context of the three datasets described above.

**Two-tower model** The two-tower model [20] used two BERT Encoders, one Encoder encodes the representation of each token, and the other Encoder encodes the natural language form of the BIO tag of the label (or describes the label with other text) to obtain the label representation, and then finds the similarity between each token in the text to be predicted. The similarity between each token and all the label representations in the text to be predicted is then found, and the label with the highest similarity is found.

**BERT-CRF** The BERT-CRF end-to-end deep learning model does not require hand-motion features, and the word embedding is obtained by pre-training of BERT + fine-tune. The CRF layer only borrows the concept of transfer matrix from traditional CRF, which is completely different from traditional CRF.

**TemplateNER** TemplateNER [2] a template-based prompt method. By constructing a template for each class, it allows querying of each span of each class separately. The score of each query is obtained by generating a pre-trained LM and BART calculates the generalization probability of the query statement.

**EntLM** EntLM [14] discards the template and uses NER as a language modeling task, with the location of entities predicted as label word, and the non-entity location predicted as the original word, which is faster.

The comparison of the results between the models in this paper and the baseline models clearly demonstrates the superior performance of the proposed model, particularly when combined with the CRF decoder. The results show that the model in this paper outperforms the baseline models on both small and multiple sample datasets.

Furthermore, the stability of the performance of the proposed model on multiple sample datasets indicates its robustness and reliability. It demonstrates that the model is able to consistently achieve high performance across different datasets, suggesting its capability to generalize well and handle diverse data distributions.

Overall, the results clearly demonstrate that the model in this paper, especially when combined with the CRF decoder, exhibits superior performance on both small and multiple sample datasets. This highlights the effectiveness and stability of the proposed model for entity recognition tasks, further reinforcing its value and potential for practical applications. It can be found that the models with the CRF decoder added perform better than the model without it. We speculate that this is because our model experimented on Chinese datasets, which will have more cases of multiple tokens representing the same word compared to English data, but the model focuses more on the label of each token, and CRF alleviates this problem by focusing on the connection between tokens.

**Figure 9:**
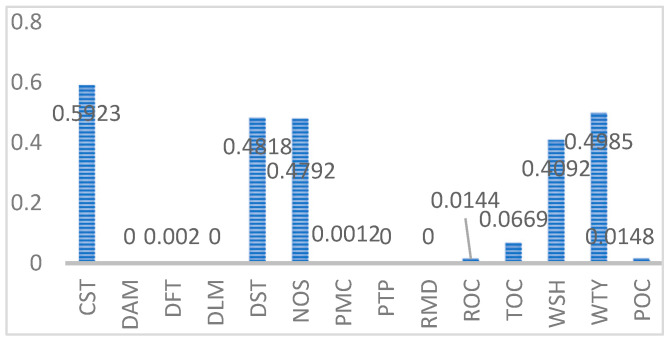
F-1 values for different types of entities.

Meanwhile, the F-1 values for different types of entities are shown in Figure 9. We can find that CST, DST, NOS, WTY, WSH-type entities have higher F-1 values. For the CST-, DST-type entities, there are more types and numbers of entities, so the training effect is better. However, for the same type and number of DST-type entities, the performance is not good because the DST-type entities are generally longer and there is no way to achieve a good result with a small amount of training data. For NOS, WTY, and WSH-type data, there are fewer entity types, and all entities are covered in the training data, so the training results are better. However, for PTP-type entities, although the entity types are also relatively few, the types involved in the training dataset are limited, so that the F-1 value is relatively low.

**Figure 10:**
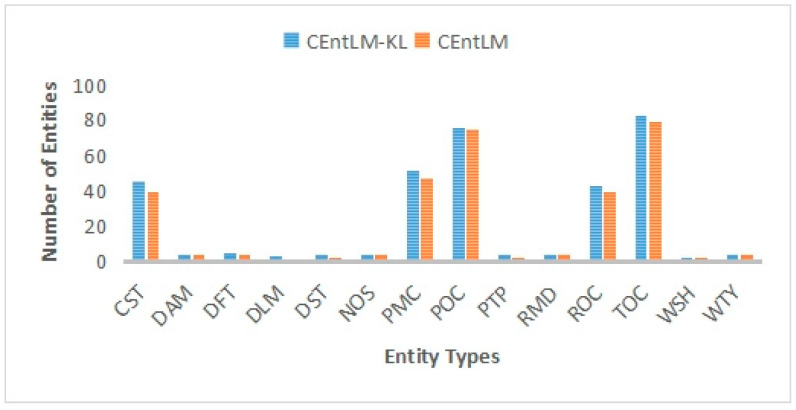
Comparison of Entity Extraction Results between CEntLM and CEntLM-KL Models.

In addition, we also compare the entity extraction results of CEntLM and CEntLM-KL, as shown in Figure 10. The comparison results in Figure 10 show that the number of entities extracted using the CEntLM-KL model is more than the number of entities extracted using the CEntLM model. By comparing the experimental results of the two models, we further proved the effectiveness of the CEntLM-KL model in practical applications.

### 3.3. MPD Knowledge Graph Construction

The data used to construct the knowledge graph in this study were obtained through a comprehensive collection and organization process. Various sources were utilized, including relevant literature on the Internet, information from reputable websites, and drilling data. The data are unlabeled data that are different from the training data. The aim was to collect a comprehensive set of data related to the drilling control domain.

**Figure 11:**
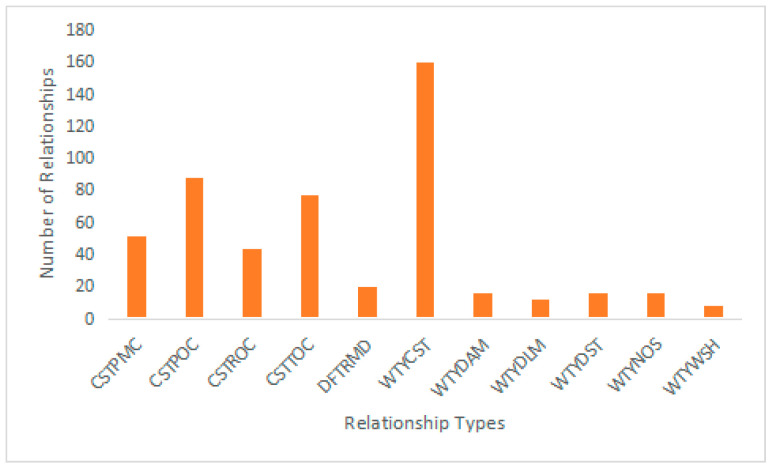
Number of Relationships.

Considering the limited availability of data, a small-scale knowledge graph was created by extracting entities and relationships. This knowledge graph includes 332 entities and 507 relationships, these are shown in Figure 10 and Figure 11. The entities represent the various elements and concepts in the drilling control domain, while the relationships are the connections and associations between these entities.

The construction of the knowledge graph involved a meticulous process of data extraction, integration, and organization. The collected data were carefully analyzed and structured to ensure the accuracy and consistency of the knowledge map. The resulting knowledge map becomes a valuable resource for understanding and exploring the field of drilling control.

Firstly, entities are extracted from the data using an entity extraction framework that combines the Trie tree method and the CEntLM-KL model. Then, rule-based methods are used for relationship extraction, with two main rules developed: Entities Word-Based Extraction and Keyword-Based Extraction. After extracting entities and relationships, the MPD knowledge graph was stored and visualized in Neo4J.

Although the knowledge graph in this study is relatively small in scale, it still provides a solid foundation for further research and analysis. It can be used as a reference and starting point for future knowledge map expansion and refinement. In addition, the insights and findings derived from this small knowledge map can contribute to a deeper understanding of the drilling control field and facilitate decision-making in related industries.

### 3.4. Knowledge Graph Reasoning

**Figure 12:**
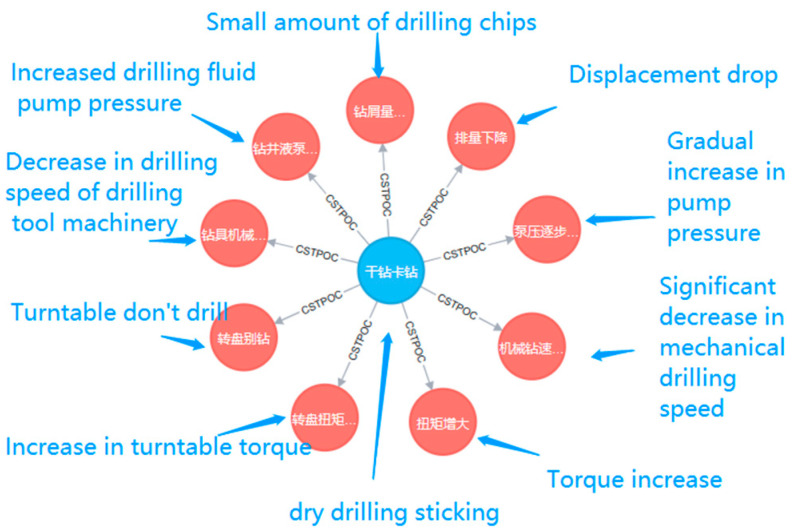
One-hop Neo4J query results.

**Figure 13:**
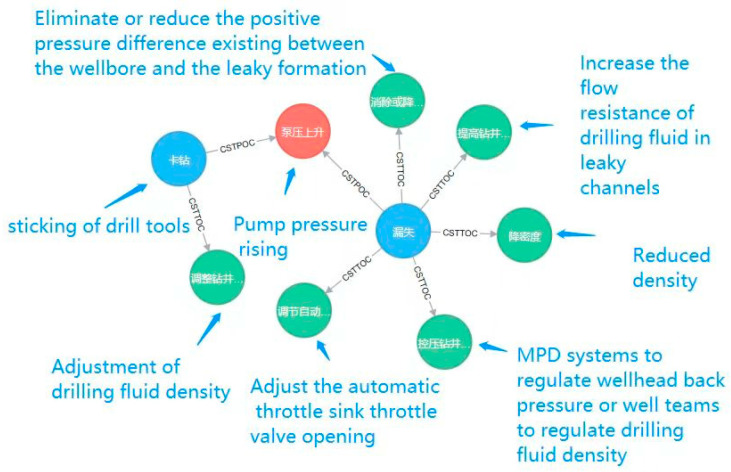
Multi-hop Neo4J query results.

The knowledge graph in the MPD domain can give possible reasons or solutions through knowledge graph reasoning when complex working conditions occur. Therefore, it is of great practical significance to construct the MPD domain knowledge graph and use it for reasoning. Timely diagnosis and prompt handling of complex working conditions can greatly reduce losses. As a result, the reasoning of MPD knowledge graph is of great significance for handling of complex working conditions. It is crucial to understand the phenomenon, cause, and treatment measures of a complex working condition. In this paper, we provide a one-hop reasoning example of the possible phenomenon of “dry drilling sticking” in reasoning as shown in Figure 12. Besides, promptly diagnosing of complex operating conditions and conducting well control are very important when an abnormal phenomenon occurs. A multi-hop reasoning example is provided in this paper for obtaining measures for dealing with “pump pressure rising” as shown in Figure 13.

#### 3.4.1. One-Hop Reasoning

Figure 12 shows the results of a query using the Neo4j query language to retrieve all nodes that have a “CST-POC” relationship with a CST-type node named “dry drilling sticking” (“干钻卡钻”). Corresponding translations are shown in Table 6. Using the query command “MATCH (n:CST)-[r:CSTPOC]->(nn:POC) WHERE n.name = ‘干钻卡钻’ RETURN nn” yields results that allow us to explore the potential outcomes or impacts when dry drill jams occur.

The results shown in Table 4 describe the nodes associated with the “CST-POC” relationship and the identified anomalies (POC) associated with the CST node “dry drill jam”. These results provide insight into specific anomalies that may arise due to dry drill jamming, allowing researchers and domain experts to understand the potential challenges and impacts associated with this particular scenario.

Targeted exploration and analysis of the knowledge graph is possible using Neo4j’s query language, with the ability to retrieve specific nodes based on defined relationships and node attributes. This capability enhances the ability to discover valuable insights and patterns in the knowledge graph, facilitating informed decision making and proactive management of drilling operations.

In summary, the Neo4j query language provides a powerful tool for querying nodes that have a specific relationship to a given node. This knowledge in the context of this example query helps to understand the potential consequences and impacts of the queried events and supports the development of effective strategies to address and mitigate these challenges in drilling operations.

#### 3.4.2. Multi-Hop Reasoning

Figure 13 shows the results of retrieving all nodes that have a “CST-POC” relationship with a node of type POC named “泵压上升” (pump pressure rising) and the results of nodes with “CST-TOC” relationship with the results obtained from the previous query are further queried. using the Neo4j query language. The corresponding translation results are shown in Table 7. In other words, we query the abnormal operating conditions that may occur when the pump pressure rises and the measures to deal with them. Using the query command “match (na:CST)-[re:CSTPOC]->(nb:POC) where nb.name = ‘泵压上升’ WITH na,re,nb match (na:CST)-[re2:CSTTOC]->(nc:TOC) return na,re,nb,re2,nc“, the results obtained allow us to explore what should be done to avoid losses to the greatest extent possible when there is a rise in pump pressure.

### 3.5. Further Analysis of Experimental Results

According to Table 4 and Figure 9, we can find that the number of various entities in the MPD Dataset are limited, mainly due to the fact that the MPD Dataset used in this paper is built manually, mainly coming from some drilling data and Internet data, and some types of entities are indeed relatively small in number, such as NOS and RMD, etc. The collected content is currently relatively small, and future improvements to the database will also be considered.

Figure 9 shows the F-1 values for different types of entities. It can be observed that there is a significant difference in F-1 values among different entities, and some entities have very low F-1 values. Through analysis, we believe that there are two main reasons.

(1) The number of entities of this type in the training set is too small, and the number of occurrences is too small to achieve good training results. For example, DAM, DFT, DLM, etc.
(2) This type of entity is generally longer and has poor training effectiveness. For example, ROC, POC, etc. For example, the ROC-type entity” 井内泥浆静止时间过长, 触变性很大, 下钻时又不分段循环, 破坏泥浆的结构”, which means“ The mud in the well is stationary for too long and thixotropic, and is not circulated in sections when drilling, which destroys the structure of the mud”.

## 4. Conclusions

MPD is the most effective means to ensure safe drilling, and MPD can avoid further deterioration of complex conditions through fine control of wellhead back pressure. The key to successful MPD is the control strategy, but the current well control strategy relies strongly on manual experience, which hinders the automation and intelligence of well control. To address this problem, this paper constructs an MPD knowledge graph, which extracts knowledge from published papers and drilling reports to guide well control. To improve the performance of entity extraction in this knowledge graph, this paper extends the EntLM model to a few-shot Chinese entity recognition model CEntLM-KL and constructs KL entropy to enhance the entity recognition accuracy. By experimenting on the standard dataset, it is shown that the method proposed in this paper has significantly improved compared with the SOTA method, and the entity recognition F-1 value reaches 33% on the drill-down dataset with small samples. Finally, this paper stores the knowledge graph into Neo4J and performs knowledge inference applications. Due to the manual construction of the domain database used in this article, the data source and volume still need to be improved. In the future, we will consider improving the MPD dataset. At present, the source of MPD knowledge is mainly the Internet and CNKI related literature as well as relevant owned MPD data, and the fusion of knowledge graph has not been considered. Our next step will be to consider fusing of the relevant knowledge of known graphs.

## Figures and Tables

**Table 1:** Types of entities.

Entity	Abbreviation	Example
Complex working conditions in the drilling	CST	Overflow, well leak
Diagnosis methods for abnormal drilling conditions	DAM	Human judgment, expert system
Drilling fluid type	DFT	Water-in-oil emulsion drilling fluid
Method of drilling a well	DLM	Underbalanced drilling
Stages of the drilling process	DST	Drilling, well cementing
Number of drillings starts during drilling	NOS	First drilling, second drilling
Abnormal conditions processing measures	PMC	Forced drilling start, reduce stress agitation
Types of pumps	PTP	Filling pumps
Drilling fluid rheological model	RMD	Power law, Newton
Reasons for complex conditions	ROC	Personnel errors, well wall instability
Treatment for abnormal conditions	TOC	Plugging of leaks, stop drilling and circulation
The shape of the well	WSH	Horizontal wells, straight wells
Types of wells	WTY	Development Wells, evaluation wells
Phenomena of abnormal conditions	POC	Pump pressure rising, torque increases

**Table 2:** Relationships based on entity word extraction.

Entity Word 1	Entity Word 2	Relationship
WTY	WSH	WTY-WSH
WTY	DST	WTY-DST
WTY	CST	WTY-CST
WTY	NOS	WTY-NOS
WTY	DAM	WTY-DAM
WTY	DLM	WTY-DLM
DFT	RMD	DFT-RMD

**Table 3:** Relationships based on keyword extraction.

Keyword	Relationship
‘特征’ (Features)	CST-POC
‘发生’ (Happen)	CST-POC
‘发现’ (Discover)	CST-POC
‘由于’ (Due to)	CST-ROC
‘造成’ (Causing)	CST-ROC
‘引起’ (Cause)	CST-ROC
‘采取’ (Take)	CST-TOC
‘应’ (Should)	CST-TOC
‘提高’ (Increase)	CST-TOC
‘减小’ (Decrease)	CST-TOC
‘进行’ (Performing)	CST-TOC
‘采用’ (Adopted)	CST-TOC
‘采用’ (Adopted)	CST-PMC
‘采取’ (Take)	CST-PMC
‘应’ (Should)	CST-PMC
‘提高’ (Increase)	CST-PMC
‘减小’ (Decrease)	CST-PMC
‘进行’ (Performing)	CST-PMS

**Table 4:** Composition of the People’s Daily-NER Dataset.

Datasets	Type	Number
People’s Daily-NER Dataset	PER	1W+
	LOC	2W+
	ORG	1W+
MPD Dataset	CST	780
	DAM	7
	DFT	13
	DLM	5
	DST	480
	NOS	92
	PMC	51
	PTP	48
	RMD	5
	ROC	62
	TOC	278
	WSH	12
	WTY	10
	POC	198

**Table 5:** F-1 scores of different models on three datasets.

Methods	People’s Daily-NER Dataset	Few-Shot-NER Dataset	MPD Dataset
Two-tower	0.9102	0.2893	0.2400
BERT-CRF	0.9125	0.2015	0.1825
TemplateNER	0.8942	0.2945	0.2545
EntLM	0.9041	0.3373	0.2999
EntLM+CRF	0.9039	0.3542	0.3022
CEntLM-KL(ours)	0.9040	0.3935	0.3215
CEntLM-KL+CRF (ours)	**0.9127**	**0.4100**	**0.3342**

**Table 6:** The phenomenon of dry drill jamming.

Entity	Phenomenon
‘干钻卡钻’ (Dry drilling sticking)	‘钻屑量小’ (Small amount of drilling chips)
	‘钻具机械钻速下降’ (Decrease in drilling speed of drilling tool machinery)
	‘排量下降’ (Displacement drop)
	‘泵压逐步上升’ (Gradual increase in pump pressure)
	‘转盘别钻’ (Turntable don’t drill)
	‘钻井液泵压增大’ (Increased drilling fluid pump pressure)
	‘机械钻速下降明显’ (Significant decrease in mechanical drilling speed)
	‘扭矩增大’ (Torque increase)
	‘转盘扭矩增加’ (Increase in turntable torque)

**Table 7:** Measures to deal with the abnormal operating conditions that may occur.

Phenomenon	One-Hop Abnormal Conditions	Two-Hop Measure
‘泵压上升’ (Pump pressure rising)	‘卡钻’ (sticking of drill tools)	‘调整钻井液密度’ (Adjustment of drilling fluid density)
	‘漏失’ (Loss)	‘消除或降低井筒与漏层之间存在的正压差’ (Eliminate or reduce the positive pressure difference existing between the wellbore and the leaky formation)
		‘提高钻井液在漏失通道中的流通阻力’ (Increase the flow resistance of drilling fluid in leaky channels)
		‘降密度’ (Reduced density)
		‘控压钻井系统调节井口回压或井队调节钻井液密度’ (MPD systems to regulate wellhead back pressure or well teams to regulate drilling fluid density)
		‘调节自动节流管汇节流阀开度’ (Adjust the automatic throttle sink throttle valve opening)

## Data Availability

Not applicable.

## References

[1] Dai Z., Wang X., Ni P., Li Y., Li G., Bai X. (2019). Named entity recognition using BERT BiLSTM CRF for Chinese electronic health records. Proceedings of the 2019 12th International Congress on Image and Signal Processing, Biomedical Engineering and Informatics (CISP-BMEI).

[2] Cui L., Wu Y., Liu J., Yang S., Zhang Y. (2021). Template-Based Named Entity Recognition Using BART. Proceedings of the Findings of the Association for Computational Linguistics: ACL-IJCNLP 2021.

[3] Fritzler A., Logacheva V., Kretov M. Few-shot classification in named entity recognition task. Proceedings of the 34th ACM/SIGAPP Symposium on Applied Computing.

[4] Li J., Chiu B., Feng S., Wang H. (2022). Few-Shot Named Entity Recognition via Meta-Learning. IEEE Trans. Knowl. Data Eng..

[5] Ma T., Jiang H., Wu Q., Zhao T., Lin C.-Y. (2022). Decomposed Meta-Learning for Few-Shot Named Entity Recognition. Proceedings of the Findings of the Association for Computational Linguistics: ACL 2022.

[6] Chen J., Liu Q., Lin H., Han X., Sun L. (2022). Few-shot Named Entity Recognition with Self-describing Networks. Proceedings of the 60th Annual Meeting of the Association for Computational Linguistics (Volume 1: Long Papers).

[7] Brown T., Mann B., Ryder N., Subbiah M., Kaplan J.D., Dhariwal P., Neelakantan A., Shyam P., Sastry G., Askell A. (2020). Language models are few-shot learners. Adv. Neural Inf. Process. Syst..

[8] Schick T., Schütze H. (2021). Exploiting Cloze-Questions for Few-Shot Text Classification and Natural Language Inference. Proceedings of the 16th Conference of the European Chapter of the Association for Computational Linguistics.

[9] Schick T., Schütze H. (2021). It’s Not Just Size That Matters: Small Language Models Are Also Few-Shot Learners. Proceedings of the 2021 Conference of the North American Chapter of the Association for Computational Linguistics: Human Language Technologies.

[10] Shin T., Razeghi Y., Logan R.L., Wallace E., Singh S. (2020). AutoPrompt: Eliciting Knowledge from Language Models with Automatically Generated Prompts. Proceedings of the 2020 Conference on Empirical Methods in Natural Language Processing (EMNLP).

[11] Gao T., Fisch A., Chen D. (2021). Making Pre-trained Language Models Better Few-shot Learners. Proceedings of the 59th Annual Meeting of the Association for Computational Linguistics and the 11th International Joint Conference on Natural Language Processing (Volume 1: Long Papers).

[12] Liu X., Zheng Y., Du Z., Ding M., Qian Y., Yang Z., Tang J. (2021). GPT understands, too. arXiv.

[13] Quimbaya A.P., Múnera A.S., Rivera R.A.G., Rodríguez J.C.D., Velandia O.M.M., Peña A.A.G., Labbé C. (2016). Named entity recognition over electronic health records through a combined dictionary-based approach. Procedia Comput. Sci..

[14] Ma R., Zhou X., Gui T., Tan Y., Li L., Zhang Q., Huang X. (2021). Template-free prompt tuning for few-shot NER. arXiv.

[15] Wiseman S., Shieber S., Rush A. (2018). Learning Neural Templates for Text Generation. Proceedings of the 2018 Conference on Empirical Methods in Natural Language Processing.

[16] Wang D., Chen Z., He W., Zhong L., Tao Y., Yang M. (2021). A Template-guided Hybrid Pointer Network for Knowledge-based Task-oriented Dialogue Systems. Proceedings of the 1st Workshop on Document-Grounded Dialogue and Conversational Question Answering (DialDoc 2021).

[17] Gomes J., de Mello R.C., Ströele V., de Souza J.F. (2022). A hereditary attentive template-based approach for complex Knowledge Base Question Answering systems. Expert Syst. Appl..

[18] Wang D., Liang Y., Xu D., Feng X., Guan R. (2018). A content-based recommender system for computer science publications. Knowl.-Based Syst..

[19] Guan R., Zhang H., Liang Y., Giunchiglia F., Huang L., Feng X. (2020). Deep feature-based text clustering and its explanation. IEEE Trans. Knowl. Data Eng..

[20] Ma J., Ballesteros M., Doss S., Anubhai R., Mallya S., Al-Onaizan Y., Roth D. (2022). Label Semantics for Few Shot Named Entity Recognition. Proceedings of the Findings of the Association for Computational Linguistics: ACL 2022.

